# Single-cell transcriptomics of pediatric Burkitt lymphoma reveals intra-tumor heterogeneity and markers of therapy resistance

**DOI:** 10.1038/s41375-024-02431-3

**Published:** 2024-10-18

**Authors:** Clarissa Corinaldesi, Antony B. Holmes, Gaia Martire, Anna Tosato, Domenico Rizzato, Federica Lovisa, Ilaria Gallingani, Qiong Shen, Lavinia Ferrone, Marian Harris, Kimberly Davies, Luca Molinaro, Umberto Mortara, Angelo Paolo Dei Tos, Kenneth Ofori, Emanuele S. G. D’Amore, Roberto Chiarle, Bo Ngan, Elisa Carraro, Marta Pillon, Shafinaz Hussein, Govind Bhagat, Marco Pizzi, Lara Mussolin, Katia Basso

**Affiliations:** 1 Institute for Cancer Genetics, Columbia University, New York, NY USA; 2 Maternal and Child Health Department, University-Hospital of Padova, Padova, Italy; 3 Istituto di Ricerca Pediatrica Citta’ della Speranza, Padova, Italy; 4 Department of Pathology, Boston Children’s Hospital, Harvard Medical School, Boston, MA USA; 5 Dana-Farber Cancer Institute, Boston, MA USA; 6 Department of Medical Science, University of Torino, Torino, Italy; 7 General Pathology and Cytopathology Unit, Department of Medicine-DMED, University-Hospital of Padova, Padova, Italy; 8 Department of Pathology & Cell Biology, Columbia University, New York, NY USA; 9 Department of Pathology, San Bortolo Hospital, Vicenza, Italy; 10 Department of Molecular Biotechnology and Health Sciences, University of Torino, Torino, Italy; 11 European Institute of Oncology IRCCS, Division of Hematopathology, Milan, Italy; 12 Hospital for Sick Children (SickKids), Toronto, ON Canada; 13 Department of Pathology, Icahn School of Medicine at Mount Sinai, New York, NY USA; 14 Herbert Irving Comprehensive Cancer Center, Columbia University, New York, NY USA

**Keywords:** Follicular B cells, B-cell lymphoma, Cancer genomics

## Abstract

Burkitt lymphoma (BL) is the most frequent B-cell lymphoma in pediatric patients. While most patients are cured, a fraction of them are resistant to therapy. To investigate BL heterogeneity and the features distinguishing therapy responders (R) from non-responders (NR), we analyzed by single-cell (sc)-transcriptomics diagnostic EBV-negative BL specimens. Analysis of the non-tumor component revealed a predominance of immune cells and a small representation of fibroblasts, enriched in NR. Tumors displayed patient-specific features, as well as shared subpopulations that expressed transcripts related to cell cycle, signaling pathways and cell-of-origin signatures. Several transcripts were differentially expressed in R versus NR. The top candidate, Tropomyosin 2 (TPM2), a member of the tropomyosin actin filament binding protein family, was confirmed to be significantly higher in NR both at the transcript and protein level. Stratification of patients based on TPM2 expression at diagnosis significantly correlated with prognosis, independently of *TP53* mutations. These results indicate that BL displays transcriptional heterogeneity and identify candidate biomarkers of therapy resistance.

## Introduction

Burkitt lymphoma (BL) is the most frequent type of B cell lymphoma in pediatric patients, accounting for 80% of the cases [1, 2]. Childhood BL includes sporadic and endemic variants, the latter geographically related to regions of high malaria prevalence. Epstein-Barr virus (EBV) infection is detected almost invariably in the endemic cases and in about 20–30% of sporadic cases [2–4]. All BL variants are characterized by uniform morphologic and immuno-phenotypic features, including monomorphic sheets of B cells with round nuclei and numerous mitotic figures intermingled with macrophages containing apoptotic debris (imparting a starry sky pattern) [2].

BL originates from the malignant transformation of B cells in the germinal centers (GC), which represent the sites of antibody affinity maturation, a process based on multiple rounds of B cell receptor (BCR) editing by somatic hypermutation (SHM) followed by affinity-driven selection [5]. The GC includes two histologically and functionally distinct compartments, the dark zone (DZ), where B cells proliferate and undergo SHM, and the light zone (LZ) where cells undergo selection based on the affinity of their receptors for the antigen [6, 7]. Recent single cell (sc)-transcriptomic analyses showed that GC B cells are more likely to move along a continuum of states rather than between two defined and uniform populations [8–11]. The cell-of-origin of BL is considered to be a GC B cell at the DZ stage of differentiation [12, 13].

Translocations involving the *MYC* locus on chromosome 8 (8q24) and the loci encoding the heavy and light chain of the immunoglobulin receptor represent the genetic hallmark of BL [2, 3, 14, 15]. The result of these translocations is the de-regulated ectopic expression of MYC in GC B cells [5, 16, 17]. Although invariably present, ectopic expression of MYC is not sufficient for lymphomagenesis and a number of cooperating genetic lesions have been identified, including those leading to aberrant activation of the PI3K signaling pathway, stabilization of CCND3, constitutive activation of TCF3 by targeting both *TCF3* and its negative modulator *ID3*, *TP53* inactivation, and disruption of the Gα13-dependent GC B cell confinement pathway [2]. Overall, these observations suggest that the malignant transformation process relies on hijacking pathways that are essential for GC B cell physiology.

It remains an open question whether BL includes distinct molecular subtypes: transcriptomic analyses have confirmed the distinct profile of BL, although differences across variants appear modest [12, 18–20]. More recently, three subgroups were identified, based on genetic features, in a dataset including both adult and pediatric BL [21]. Genetic characterization of endemic and sporadic BL variants showed that EBV-positive BLs, regardless of the geographic origin, are associated with higher expression of AICDA and increased aberrant SHM-driven mutational burden, but with fewer mutations in driver genes such as the *TCF3*/*ID3* module, *CCND3* and *TP53* [19, 21, 22]. Based on these observations, the EBV status appears a more relevant criterion for BL classification than the geographic location.

Treatment with dose-intensive chemotherapy associated to intense supportive care is effective in curing more than 90% of pediatric BL patients. However, no effective treatment is available in the case of recurrent or refractory disease [23]. Except for *TP53* alterations, which have been associated with poor prognosis [24–27], the overall genetic and epigenetic traits affecting the BL transcriptome in non-responder (NR) versus responder (R) patients remain to be elucidated. In order to address this critical issue, we have used sc-transcriptomic analysis to investigate BL intra-tumor heterogeneity, its relationship with normal GC B cell transcriptional programs, and changes that correlate with outcome.

## Materials and methods

### Ethics approval

This research has been performed in accordance with the Declaration of Helsinki. All patients or guardians provided informed consent. The study was approved by the Ethic committee at the University-Hospital of Padova (Comitato etico per la sperimentazione clinica della provincia di Padova) on May 5th, 2022 (reference number 5077/ao/21) and by the Columbia University Human Research Protection Office & Institutional Review Board (Protocol number AAAS3727).

### Tumor specimens

Specimens for sc-transcriptomics (11 patients), qRT-PCR (extension set, *n* = 57), and immunohistochemistry (IHC, *n* = 11) were obtained from EBV-negative pediatric BL patients enrolled in the AIEOP LNH-97 protocol [28]. An independent cohort (36 patients), used only for IHC was retrieved from the archives of the Departments of Pathology at Columbia University Irving Medical Center (New York, NY, USA), at the Dana-Farber Cancer Institute (Boston, MA, USA), at the University of Torino (Torino, Italy), and at the Hospital for Sick Children (SickKids, Toronto, ON, Canada) (Supplementary Table 1 and Supplementary Materials and Methods).

### Single-cell gene expression and V(D)J profiling

Sc-transcriptomics was performed using the Chromium Next GEM Single Cell 5’ kit v2, the Chromium Next GEM Single Cell V(D)J Enrichment Human B cell Kit, and the Chromium Controller (10x Genomics), following the manufacturer’s instructions. Sequencing was performed on the NovaSeq 6000 System (Illumina). Further details are available in the Supplementary Materials and Methods.

### Gene set and pathway enrichment analysis

Pathway enrichment analysis was performed using a one-sided hypergeometric test assessing P(X ≥ N) with a Benjamini-Hochberg false discovery rate correction on the KEGG (c2.cp.kegg.v6.2), BioCarta (c2.cp.biocarta.v6.2), and Hallmark (h.all.v7.0) collections from the Molecular Signature Database (MSigDB) v6.2 (http://software.broadinstitute.org/gsea/msigdb/index.jsp) [29] or the SignatureDB database (https://lymphochip.nih.gov/signaturedb/).

### qRT-PCR

Total RNA was isolated using Trizol reagent and retrotranscribed with SuperScript II reverse transcriptase (ThermoFisher Scientific). qRT-PCR was performed using Fast SYBR^TM^ Green Master Mix (ThermoFisher Scientific). The relative expression levels were calculated according to the comparative delta CT (threshold cycle number) method (2^−ΔΔCt^), using *GAPDH* as housekeeping gene. Primers are reported in the Supplementary Table 2.

### TP53 mutational analysis

Mutational analyses were performed on exons 5, 6, 7, and 8 of *TP53* gene by PCR amplification and sequencing (Supplementary Materials and Methods).

### Immunohistochemistry

Formalin-fixed and paraffin-embedded (FFPE) tumor or reactive lymphoid tissue 3 μm-thick sections were used for immunohistochemical staining using anti-TPM2 antibody (1:200, rabbit, Proteintech, 11038-1-AP Lot#94880) and standard procedures (Supplementary Materials and Methods).

TPM2 expression in BL cases was considered positive when >30% tumor cells showed clear-cut cytoplasmic expression, irrespective of staining intensity. The scoring was performed in sections with reliable internal positive (i.e. blood vessel and/or muscle tissue) and negative (i.e. GC B cells, adipocytes, collagen fibers) controls.

### Statistical analysis

The MAST R package [30] was used to identify differentially expressed genes in each cluster. For the gene set and pathway enrichment analysis, a hypergeometric test with a Benjamini-Hochberg false discovery rate correction was used. Mann-Whitney *U* Tests were performed using the Python SciPy scipy.stats.mannwhitneyu package.

Survival analyses were performed using the Kaplan-Meier method with the Lifelines Python package; the logrank.test function was applied to assess the significance of the survival curves.

Detailed information of the statistical test, number of replicates/samples (defined as n) used in each experiment, and measurement precision are reported in the figure legends. Significance was associated to a *p* < 0.05.

## Results

### Heterogeneity of the single-cell transcriptome of BL tumors

We performed paired sc-transcriptomic and immunoglobulin repertoire analyses on 12 sporadic EBV-negative BL specimens (11 patients), including 8 pleural or abdominal effusions (E) and 4 nodal tumor masses (N), which were collected at diagnosis and frozen as viable single cell suspensions (Supplementary Fig. 1A). All patients were treated with the same therapeutic protocol (AIEOP LNH-97) [28]. The majority (8/11) responded to therapy (responders, R) and are currently in lasting remission (≥3 years). The non-responder (NR) patients experienced disease progression (BL102 and BL107) or early relapse (BL103, relapse 2 weeks after remission) and died of the disease.

Upon data quality filtering, we obtained 36,400 cells, including 21,649 tumor cells identified based on the presence of clonal V(D)J rearrangements, and 14,751 tumor-infiltrating normal cells. Each specimen displayed variable number of cells (range 838–6804) and fractions of tumor (median 62%) and non-tumor (median 38%) cells (Supplementary Fig. 1B).

V(D)J analysis identified the tumor clone carrying a clonally rearranged B cell receptor (BCR) in each patient (Supplementary Table 3). Consistent with previous reports suggesting high frequency of BCR rearrangements involving the *IGHV3* genes [22, 31], this *V* gene family was observed in 7/11 tumors with recurrent usage of *IGHV3-23* and *IGHV3-33*. The lambda light chain locus was rearranged in 7/11 patients with recurrent usage of the *IGLV2-14* and *IGLV1-51 V* genes. Cytofluorimetric data on surface expression of kappa/lambda chains were available for the diagnostic specimen of 5/11 patients and were concordant with the chains identified by sc-RNAseq analysis. Most patients (10/11) expressed exclusively unswitched IGHM and IGHD isotypes (Supplementary Table 3).

**Fig. 1:**
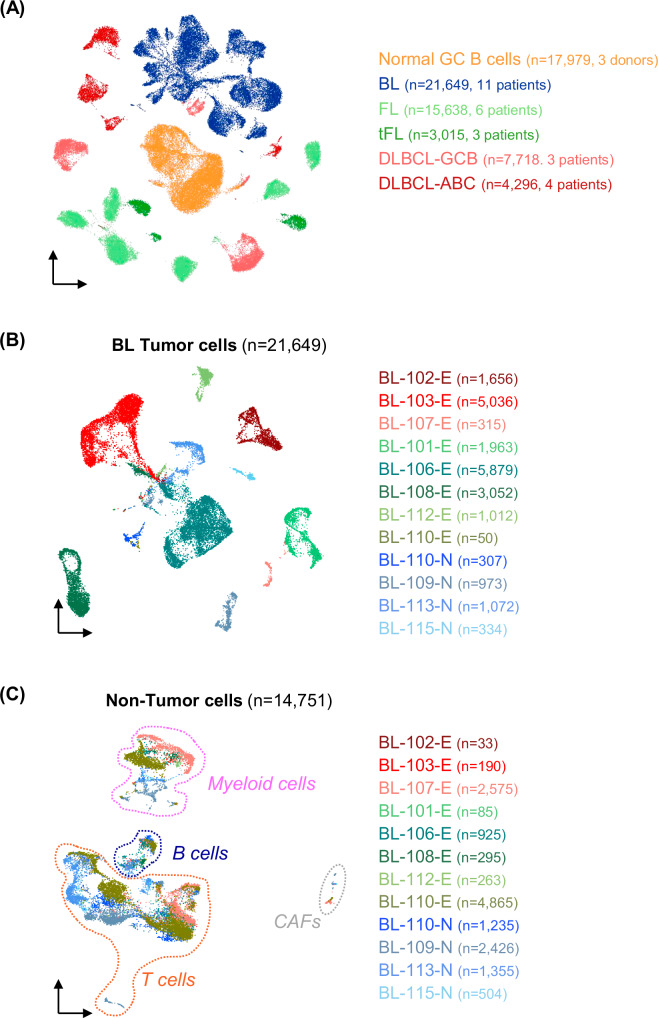
Heterogeneity in the tumor cell transcriptome of GC-derived lymphomas. UMAP projections of sc-transcriptomic profiles of: **A** normal GC B cells and tumor cells isolated from BL, follicular lymphoma (FL), transformed-FL (tFL), and diffuse large B cell lymphoma (DLBCL), including both GCB and ABC subtypes [8, 32, 33]; **B** 21,649 BL tumor cells isolated from 12 diagnostic specimens (11 patients); **C** 14,751 normal tumor-infiltrating cells isolated from the same BL specimens. Immunoglobulin gene transcripts were excluded. E Effusion, N nodal; CAFs cancer-associated fibroblasts.

Analysis of tumor cells from our BL dataset in comparison with published sc-transcriptional profiles of normal GC B cells [8], and GC-derived lymphomas including follicular lymphoma (FL), transformed FL (tFL) and diffuse large B cell lymphoma (DLBCL) [32, 33], confirmed that these tumor types are distinct and segregate separately from normal GC B cells (Fig. 1A).

Individual tumor cases were distinguishable based on transcriptional features of the malignant cells in all tumor types, including BL (Fig. 1B and Supplementary Fig. 1C). Conversely, the same analysis performed on the non-tumor cells in the BL specimens (*n* = 14,751) revealed that cells from different patients intermingled and clustered by cell type rather than by specimen (Fig. 1C). These observations indicate that differences across patients are mostly driven by transcriptional features associated with the tumor cells.

To investigate the inter-tumor features driving the patient-specific BL profiles, we first identified the most differentially expressed genes in the tumor cells of each patient when compared to all the others. Then we performed pathway enrichment analysis on the top 100 upregulated genes in each patient using the SignatureDB database. The results showed that some transcriptional programs including those modulated by MYC and BCL6 are largely shared across patients, although each patient may display a unique subset of targets (Supplementary Fig. 1D and Supplementary Table 4). Other programs, including TCF3 targets, genes affected by signaling pathways (i.e. PI3K, CD40, interferon) or associated with proliferation were enriched in subsets of patients (Supplementary Fig. 1D and Supplementary Table 4). These results suggest that the patient-specific signatures are driven by unique transcriptional features, some of which however converge into the same pathways.

### Tumor-infiltrating normal cells comprise mostly immune cells

**Fig. 2:**
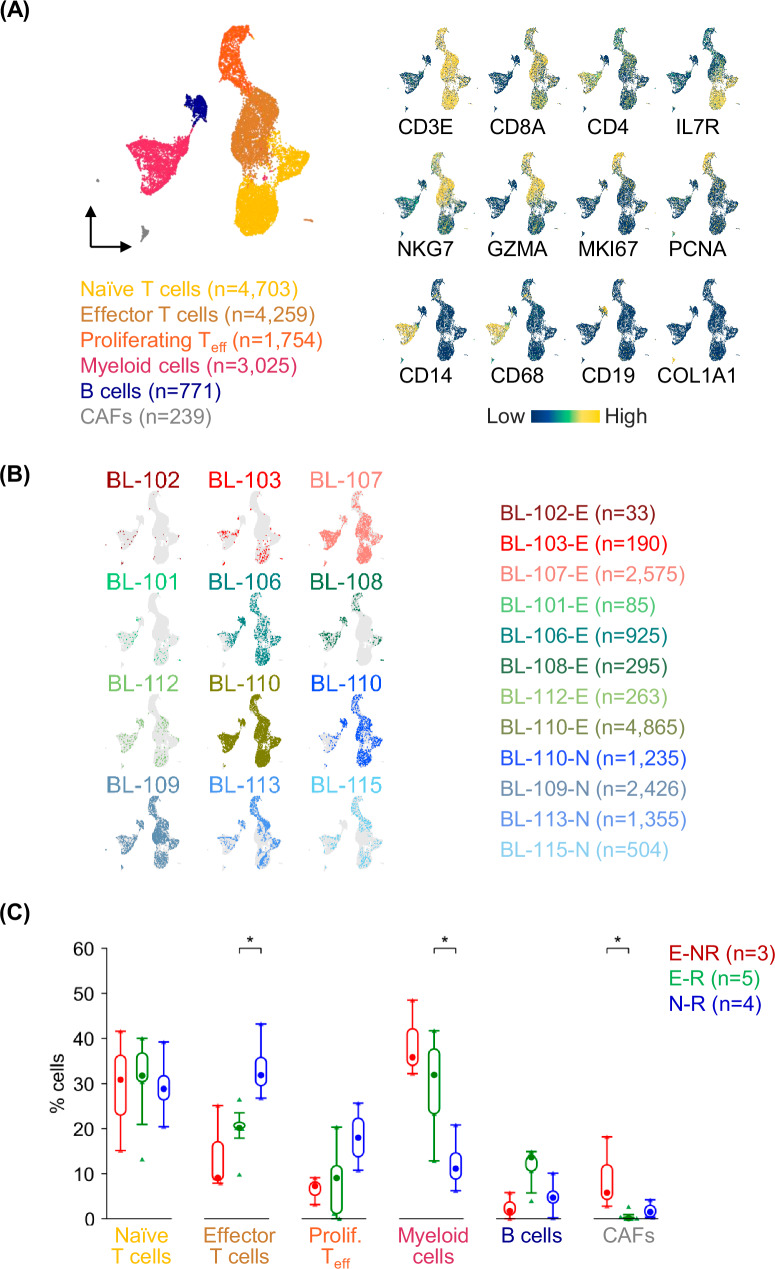
Tumor-infiltrating normal cells include multiple immune cell types. **A** UMAP projection of sc-transcriptomic profiles of 14,751 normal tumor-infiltrating cells, labeled based on the identified populations. Relative gene expression is displayed as UMAP/heatmap with colors representing the z-scored log2 normalized expression. **B** UMAP projections of normal tumor-infiltrating cells, as shown in (**A**), color-coded based on the cells contributed by each specimen. **C** Box and Whisker plots displaying the distribution of normal tumor-infiltrating cells in the dataset stratified by sample origin (E effusion, N nodal) and response to therapy (non-responders, NR, and responders, R). A Mann-Whitney *U* Test was used to compare data sets pairwise (*p < 0.05).

The analysis of non-tumor cells in the BL specimens revealed mostly immune cells including distinct clusters of naïve and effector T cells, myeloid cells, and B cells (Fig. 2A). In addition, a small fraction of fibroblasts (cancer-associated fibroblasts, CAFs) was detected (Fig. 2A). The identity of the subpopulations was determined based on the expression of specific markers and confirmed by assessing enrichment in the LM22 signatures using CIBERSORT and the Mann-Whitney-Wilcoxon Gene Set test (MWW-GST) [34] (Fig. 2A). Tumor-infiltrating normal cells were found in all specimens, although with different representation (Fig. 2B). Some subpopulations, including naïve T cells and B cells, were evenly represented across samples, while others showed a bias based on specimen type (effusion, E, vs nodal, N) or prognosis (Fig. 2C). T cells, particularly effector T cells, were more abundant in the N specimens, while myeloid cells represented a larger fraction of infiltrating cells in the E samples (Fig. 2C). Although the number of CAFs was modest, a significant enrichment (*p* < 0.05, Mann-Whitney *U* Test) was detected in the specimens from NR compared to R patients (Fig. 2C).

A refined analysis focusing on the T cell compartment increased the resolution and allowed the detection of natural killer and regulatory T cells, in addition to naïve and effector T cells (Supplementary Figure 2A). This analysis highlighted that most T cells detected in the E specimens were naïve T cells, while effector T cells were significantly enriched in the N specimens (Supplementary Fig. 2B). No significant distribution differences were detected in the natural killer and regulatory T cell compartments (Supplementary Fig. 2B). A subset of T effector cells expressed transcripts of exhaustion markers, including *LAG3*, *HAVCR2* (TIM3), and *PDCD1* (PD1), while they lacked expression of *TCF7* (Supplementary Fig. 2C). By applying a previously reported exhaustion scoring approach [35], we scored as exhausted 38% of the overall T_eff_ cells and 19% of the proliferating T_eff_ cells, mostly in the N specimens (Supplementary Fig. 2D, E).

The myeloid component showed some heterogeneity that was captured by an analysis focused on these cells (Supplementary Fig. 3A). We identified six clusters which displayed expression of markers previously reported to be associated with multiple myeloid subpopulations including classical tumor-infiltrating monocytes, inflammatory and regulatory tumor-associated macrophages, and conventional CD1c^+^ dendritic cells (Supplementary Fig. 3B) [36]. A subpopulation with monocytic features and a subpopulation of regulatory tumor-associated macrophages were shown to be respectively depleted and enriched in the N specimens (Supplementary Fig. 3C).

Although several differences seem to be driven by the biospecimen types (N vs E), these data suggest that some cellular components (i.e. CAFs) may be relevant in defining biological features associated with disease outcome (see Discussion).

### BL intra-tumor heterogeneity reflects similarities with distinct normal GC subpopulations

**Fig. 3:**
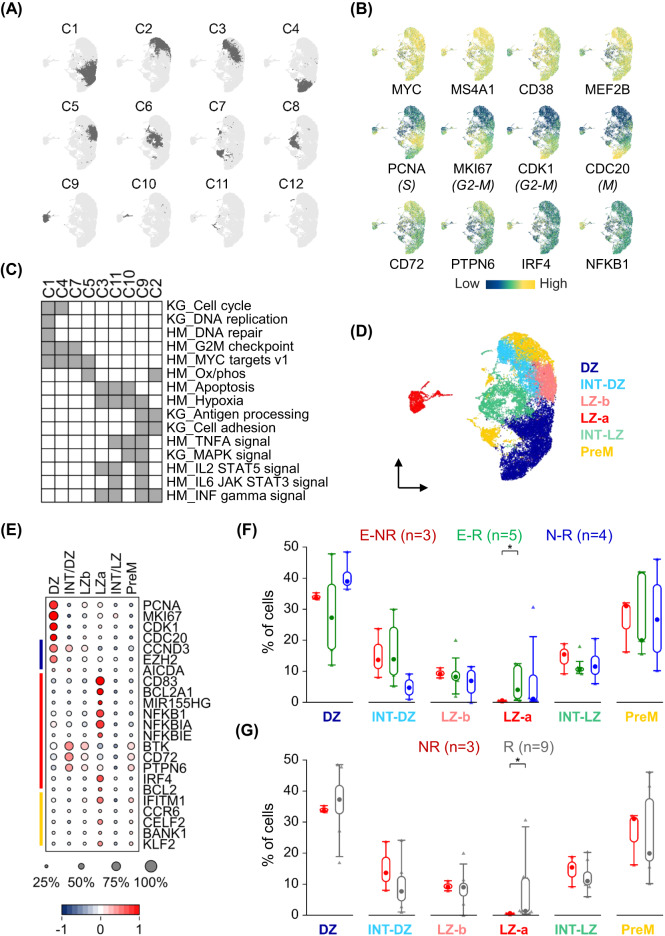
BL intra-tumor heterogeneity reflects similarities with distinct normal GC subpopulations. **A** UMAP projection and cluster identification using sc-transcriptomic profiles of 21,649 BL tumor cells. **B** Relative gene expression displayed as UMAP/heatmap with colors representing the z-scored log2 normalized expression. **C** Pathway enrichment analysis for the gene signatures (top 100 upregulated) associated with the clusters identified in (**A**). Relevant pathways from KEGG (KG) and Hallmark (HM) databases that are significantly enriched (hypergeometric test with Benjamini-Hochberg correction, *q* < 0.05) are shown in gray. **D** UMAP projection of BL tumor cells as displayed in (**A**) and colored based on the highest correlation of each cluster with previously reported signatures of normal GC B cell subpopulations [9]. DZ dark zone, INT intermediate, LZ light zone, PreM memory precursors. **E** Heat map displaying a subset of differentially expressed genes in the subgroups identified in (**D**). The color bars on the left indicate genes associated with the DZ (blue), LZ (red) or PreM (yellow) signatures. The size of the dot indicates the percentage of cells with detectable expression, and the color shows the z-scored average (log2) normalized expression within a group. Box and Whisker plots displaying the distribution across the GC-related subgroups of BL tumor cells stratified by: **F** sample origin (E effusion, N nodal); **G** response to therapy (NR non-responders, R responders). A Mann-Whitney *U* Test was used to compare data sets pairwise (*p < 0.05).

Analysis of the BL tumor sc-transcriptomes revealed distinct clusters in each patient, mostly driven by the heterogeneous expression of cell cycle markers and molecules involved in the BCR signaling pathway and NF-κB activation (Supplementary Figure 4). These features were recurrent across patients and the analysis of the merged dataset confirmed the presence of several transcriptional programs that were shared across multiple patients (Fig. 3A). As expected, expression of the *MYC* oncogene, as well as B cell and GC B cell markers was quite uniform across all cells (Fig. 3B, top). Conversely, cell-cycle markers (including *PCNA*, *MKI67*, *CDK1*, *CDC20*) discriminated clusters of cells with a transcriptome consistent with active cell division (Fig. 3B, middle) from cells expressing higher levels of transcripts related to B cell activation, BCR (*PTPN6*, *CD72*) and NF-κB (*NFKB1*, *IRF4*) pathways (Fig. 3B, bottom). Pathway enrichment analysis of the top 100 genes upregulated in each cluster using the KEGG and Hallmark databases confirmed enrichment for genes promoting cell-cycle progression (Clusters 1, 4, and 7), antigen presentation (Clusters 2, and 9), and multiple signaling pathways including MAPK, NF-κB, interleukins and interferon (Clusters 2, 3, 9, 10, and 11) (Fig. 3C and Supplementary Table 5).

Cells were distributed across clusters with no significant bias based on cellular representation and tumor specimen source (E vs N samples) (Supplementary Fig. 5A, B). Regarding patient outcome, significant depletion in the NR specimens was detected for one of the smallest clusters (cluster 10) that was associated with multiple signaling pathways, including MAPK and NF-κB (Supplementary Fig. 5C).

In order to investigate the relationship between BL cells and normal GC B cells, we measured the correlation between the gene signatures associated with the BL clusters identified here and those of normal GC B cell subpopulations that we have previously reported [9]. This approach provides a score of similarity (or dissimilarity) for each tested signature, and each population was annotated based on the best enrichment score. Bulk transcriptomic analysis of BL specimens displayed significant correlation with dark zone (DZ) GC B cells. However, at the single cell level we also identified groups of tumor cells carrying features of GC light zone (LZ), intermediate (INT) and memory B cell precursors (PreM) (Fig. 3D). Several markers that are associated with these GC B cell subpopulations displayed increased expression in distinct clusters of BL cells (Fig. 3E). BL cells resembling DZ or INT cells closer to the DZ (INT-DZ) represented about 50% of the tumor cells, while the remaining were similarly distributed between LZ-b (representing the late LZ stages), INT-LZ, and PreM groups (Fig. 3F). Conversely, cells carrying the LZ-a signature (associated with the early LZ stages) were unevenly distributed across patients and significantly enriched only in a minority of patients (3/11), suggesting that they represent a minor component of the tumor population in most cases. Of note, cells displaying the LZ-a signature were significantly depleted in the NR specimens (Fig. 3G). To identify potential state transitions, we performed pseudo-temporal analysis and showed that these different states are predicted not to be fixed, rather cells may change their transcriptional state following specific patterns (Supplementary Fig. 5D).

In conclusion, the transcriptional heterogeneity observed in BL tumor cells can be annotated along the diverse states of GC B cells suggesting that BL cells recapitulate some of the features associated with normal GC developmental stages.

### Identification of genes differentially expressed at diagnosis and correlating with therapy response

**Fig. 4:**
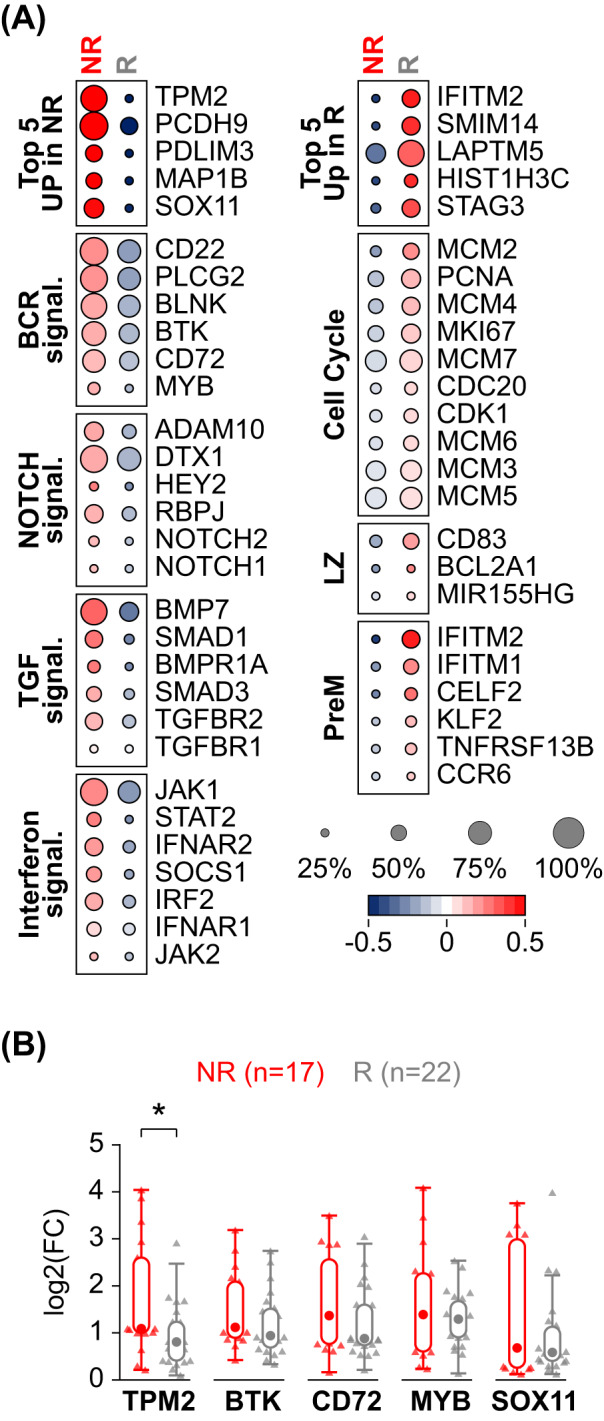
Identification of transcriptional features related to response to therapy. **A** Heat map displaying a subset of differentially expressed genes in sc-transcriptomic profiles of tumor cells from non-responders (NR) and responders (R) to therapy. The size of the dot indicates the percentage of cells with detectable expression, and the color shows the z-scored average (log2) normalized expression within a group. **B** Box and Whisker plots displaying the expression fold change of selected genes in diagnostic specimens of NR versus R, as detected by qRT-PCR. A Mann-Whitney *U* Test was used to compare data sets pairwise (*p < 0.05).

Toward the identification of markers with the potential to discriminate the NR patients at diagnosis, we performed differential expression analysis on the sc-transcriptomic data from the tumor cells of NR versus R specimens. This analysis identified 1,739 upregulated and 216 downregulated genes with a fold change >1.5 (Supplementary Table 6). The top upregulated genes in the NR group included several cytoskeleton-related transcripts (*TPM2*, *PCDH9*, *PDLIM3*, and *MAP1B*) and *SOX11* (Fig. 4A). Previous studies have shown that SOX11 is variably expressed in BL and that higher nuclear protein expression correlates with worse prognosis in adult cases [37–39]. However, its role as a prognostic feature in pediatric BL has not been explored. A number of NR-upregulated genes were involved in diverse signaling pathways, associated with BCR, NOTCH, TGF-β, and interferon (Fig. 4A). The genes upregulated in the R specimens were enriched for cell cycle genes, and for markers associated with the LZ and PreM signatures (Fig. 4A).

A few candidates including genes with a relevant function in B cells (*MYB*, *BTK*, *CD72*), *SOX11*, and tropomyosin 2 (*TPM2*), a member of the tropomyosin actin filament binding protein family [40] that was the most upregulated gene in the NR group (Fig. 4A), were selected for further validation in an extended cohort of patients. Quantitative RT-PCR on diagnostic specimens from 22 R and 17 NR showed significantly higher expression of *TPM2* in the NR compared to the R group (*p* < 0.05). All other tested candidates displayed a trend toward higher expression in the NR (Fig. 4B). Although the validation panel included unpurified N biopsies and E samples, no significant expression differences were observed considering the specimen types, confirming that the sample origin was not a variable significantly affecting this analysis.

**Fig. 5:**
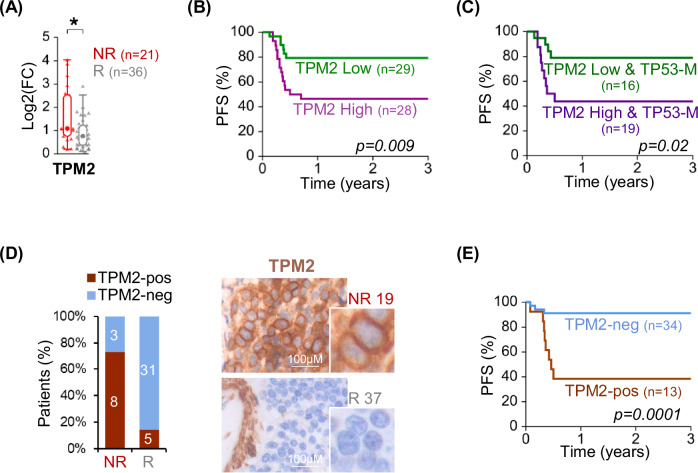
TPM2 is a prognostic biomarker in pediatric EBV-negative BL. **A** Box and Whisker plot displaying *TPM2* expression fold change in diagnostic specimens of 21 non-responder (NR) and 36 responder (R) patients, as detected by qRT-PCR. (*p < 0.05 by Mann-Whitney U Test). **B** Kaplan-Meier plot for progression free survival (PFS) analysis in the BL patients (*n* = 57) stratified based on expression of *TPM2* transcript, as detected by qRT-PCR in (**A**). “TPM2 low” and “TPM2 high” are patients with *TPM2* expression below and above the median expression in the dataset, respectively. **C** Kaplan-Meier plot for PFS analysis in the subset of patients (*n* = 35) carrying mutated *TP53* and stratified based on expression of *TPM2* transcript, as detected by qRT-PCR in (**A**). **D** Bar plot displaying the percentage of cases which scored as positive (TPM2-pos) or negative (TPM2-neg) for TPM2 protein expression, as detected by IHC analysis in 11 NR and 36 R patients. (Right) Representative images of TPM2 detection by IHC in BL nodal diagnostic biopsies from a R (R37) and a NR (NR19). TPM2 expression is detectable in the tumor cells of the NR and in the normal muscle, stroma, and macrophages of all specimens. **E** Kaplan-Meier plot for PFS analysis in BL patients (*n* = 47) stratified based on TPM2 protein expression in the tumor cells.

The expression of the top performing candidate (*TPM2*) was assessed in a further extended panel including a total of 36 R and 21 NR (Fig. 5A). Patient stratification based on qRT-PCR in the diagnostic specimens indicated that high *TPM2* transcript expression (above the median expression in the dataset) was significantly associated with poor prognosis (Fig. 5B and Supplementary Table 7). In addition, we performed mutational analysis for *TP53* in the same cohort and showed that *TPM2* expression significantly associated with progression-free survival even in the high-risk subset of patients carrying *TP53* mutations (Fig. 5C and Supplementary Table 7). All patients included in these analyses were uniformly diagnosed and treated in Italy (AIEOP LNH-97) [28].

In order to confirm expression in the tumor cells, we tested TPM2 protein by IHC in 47 BL patients (10 of which were analyzed also by sc-RNAseq and/or qRT-PCR) using diagnostic tissue samples collected at multiple Institutions in Europe, USA, and Canada. The antibody reactivity and specificity were validated by immunoblotting and IHC in HEK-293T cells transfected with a plasmid expressing an HA-tagged TPM2 protein (Supplementary Fig. 6A, B). Normal GC B cells from reactive lymphoid tissue showed no or barely detectable TPM2 expression, while expression was observed in follicular dendritic cells, macrophages and, as expected, in stromal cells, and in smooth and striated muscle cells (Supplementary Fig. 6C). Analysis of the primary BL specimens showed cytoplasmic TPM2 expression in the tumor cells of 8/11 (73%) NR but only 5/36 (14%) R patients, showing a significant association between this marker and resistance to therapy (*p* = 0.0002 by Fisher Exact test) (Fig. 5D and Supplementary Fig. 6D). In the “positive” cases, TPM2 was expressed in the large majority of tumor cells with a signal intensity varying from weak to strong across cases (Supplementary Fig. 6D). There was perfect concordance between the protein and RNA expression as detected by IHC and by sc-RNAseq, while detection by qRT-PCR and IHC was discordant in three cases. Of note, the single TPM2-positive case in the R group (BL101) for which sc-transcriptomics detected *TPM2* RNA expression in the tumor cells, indeed displayed TPM2 protein staining in the tumor cells by IHC (Supplementary Fig. 6D). Progression-free survival analysis performed by stratifying the patients based on TPM2 protein expression confirmed the significant prognostic value of this biomarker (Fig. 5E and Supplementary Table 7). Overall, these results identify TPM2 as a potential prognostic biomarker to stratify patients at diagnosis by qRT-PCR or IHC.

## Discussion

Single-cell transcriptomic analyses have been implemented in the study of normal GC B cells as well as GC-derived lymphomas, including FL and DLBCL, highlighting a previously unappreciated level of intra-tumor heterogeneity that encompasses both the malignant cells and their microenvironment [8, 9, 11, 32, 33, 41, 42]. Here, we provide the first atlas of sc-transcriptomic data for pediatric BL, confirming considerable heterogeneity in the transcriptional programs of this phenotypically uniform disease, when explored at single-cell level. The intra-tumor transcriptional heterogeneity suggests the presence of alternate states of cell division and activation of several signaling pathways that appear to mimic features of normal GC B cells transitioning from DZ to LZ stages and vice versa. These observations contrast the current view of BL tumor cells uniformly resembling DZ GC B cells. The evidence of signaling activity in several pathways suggests that tumor cells may receive stimulation from the surrounding microenvironment, an aspect that has not been extensively investigated in BL and may require future studies given its potential therapeutic implications. The observed heterogeneity may also reflect the distinct genetic make-up, which was not investigated in this study.

Although BL is a histologically uniform neoplasm, consisting of sheets of medium-sized tumor cells with scattered macrophages engulfing apoptotic debris (“starry sky pattern”) [2], our analysis detects a variety of infiltrating immune cells, some of which may be relevant to provide the stimuli that induce the changes in the BL transcriptional profiles. Indeed, we identified a large fraction of T cells, including naïve, regulatory and effector T cells, both in the nodal and effusion specimens. An unexpected observation to be further explored was the identification of a small subset of fibroblasts, the presence of which correlated with refractory/relapsed disease. Although the role of these cells in BL has not been investigated, CAFs have been shown to promote tumor relapse and therapeutic resistance in several cancer types [43, 44]. Our study includes mostly abdominal and pleural effusions; therefore, a full characterization of the normal microenvironment will require a more extensive analysis of nodal specimens.

In our comparison of diagnostic specimens from R and NR, we identified multiple molecules involved in the BCR signaling pathway with a consistent trend toward increased expression in refractory tumors. These markers should be evaluated further in purified tumor specimens, given the role of BCR signaling in BL. Indeed, BCR signaling and the downstream activation of PI3K pathway are relevant to the pathogenesis of BL [45–51]. Mutations targeting several key players, including *TCF3* and its negative regulator *ID3*, foster the constitutive activity of the BCR promoting BL cell survival [49]. In this context, a correlation between higher expression of genes in the BCR signaling pathway and refractory disease may provide a rationale for the therapeutic inhibition of this pathway in NR patients, whose treatment is still an unmet clinical need.

The identification of TPM2 as a potential prognostic biomarker and the development of a simple IHC-based assay for its detection lays the basis to include TPM2 in the current panel of markers used for BL diagnosis. Although an uncommon event, relapsed and refractory pediatric BL are incurable, and no biomarkers are currently available to identify at diagnosis patients who are unlikely to respond to therapy. TPM2 expression is shown here to inform patient stratification in a collection of 99 unique specimens provided by multiple institutions across the world, suggesting that TPM2 is a robust candidate biomarker.

In addition to TPM2, several molecules involved in cytoskeleton remodeling were identified among the most differentially expressed genes between specimens from NR and R patients. The identification of these molecules was possible because tumor cells were analyzed separately from the microenvironment, which includes cells (i.e. smooth and striated muscle cells) characterized by high levels of these markers. The cytoskeleton provides mechanical support to cells and is involved in intracellular transport, signaling cascade scaffolding, and cell migration [52–54]. The aberrant expression in BL cells from NR patients of an isoform of tropomyosin (TPM2) mostly expressed in slow muscle fibers, strongly suggests that cytoskeleton features may contribute to therapy resistance of BL. This novel observation not only provides a potential biomarker for early detection of patients at high risk of refractory disease, but also instructs on the need to investigate the role of the cytoskeleton in BL pathogenesis.

## Supplementary information

Supplementary Materials and Methods

Supplementary Figures

Supplementary Table 1

Supplementary Table 2

Supplementary Table 3

Supplementary Table 4

Supplementary Table 5

Supplementary Table 6

Supplementary Table 7

## Data Availability

Single-cell gene expression data are available from the Gene Expression Omnibus (GEO) database under accession number GSE240252.
