# Supplementary material for: Single-cell transcriptomics of pediatric Burkitt lymphoma reveals intra-tumor heterogeneity and markers of therapy resistance
Source: Leukemia. 2024 Oct 18;39(1):189–98. doi: 10.1038/s41375-024-02431-3 (PMC11717704; doi:10.1038/s41375-024-02431-3)
Supplement: Supplementary file 1 — Supplementary Materials and Methods [file 41375_2024_2431_MOESM1_ESM.pdf]

MATERIALS AND METHODS

Tumor specimens

BL diagnostic specimens, including pleural and abdominal effusions (E) and nodal tumor masses (N), were collected at diagnosis and frozen as viable single-cell suspensions. All specimens were centralized and reviewed independently by two pathologists who confirmed the BL diagnosis. All specimens were characterized for tumor cell representation at the time of collection by FACS and/or histological analysis and displayed a tumor content >60%. All patients were treated according to the AIEOP LNH-97 protocol[1] and stratified according to the St. Jude staging system[2]. Cells used for sc-transcriptomic analysis were obtained from 11 patients (9 males and 2 females), with a median age at diagnosis of 10 years (range 5-16 years). All patients were EBV-negative and the t(8;14) was successfully detected by LD-PCR and/or FISH. No other clinical or biological criteria were used to select patients.

The extension set comprised 57 EBV-negative pediatric BL patients enrolled in the AIEOP LNH-97 protocol[1], of which 6 were included in the sc-RNAseq analysis and 10 in the panel analyzed by IHC (Supplementary Table 1). Twenty-one experienced refractory disease or early relapse (PFS median time 120 days) and 36 obtained a continuous clinical remission. The t(8;14) was successfully detected by LD-PCR and/or FISH in 47/57 patients.

The independent IHC validation set (37 patients) included sections from FFPE tumor tissue obtained at diagnosis and retrieved from the archives of the Departments of Pathology at Columbia University Irving Medical Center (New York, NY, USA), at the Dana-Farber Cancer Institute (Boston, MA, USA), at the University of Torino (Torino, Italy), and at the Hospital for Sick Children (SickKids, Toronto, ON, Canada) (Supplementary Table 1). Three independent pathologists scored the results, while blinded to the group allocation. All specimens had a confirmed diagnosis of BL, were EBV-negative and carried *MYC* rearrangements, including t(8;14) (31 patients), t(2;8) (1 patient) and unspecified *MYC* rearrangements (5 patients). Patients

were treated according to the ANHL1131 or ANHL01P1 protocols and achieved lasting complete remissions, except for 6 cases with refractory/early relapsed disease.

EBV detection

EBV detection was performed by quantitative Real Time (qRT)-PCR on a ViiA7 Real Time PCR system (ThermoFisher Scientific) using primers (5'-CCCAACACTCCACCACACC-3'; 5'-TCTTAGGAGCTGTCCGAGGG-3') and a probe (5'-CACACACTACACACACCCACCCGTCTC-3') targeting the BamH-W region of the EBV genome. Alternatively, in-situ hybridization for EBV-encoded small RNAs was performed according to the manufacturer's protocol (Ventana Medical Systems Inc).

Single-cell gene expression and V(D)J profiling

Upon thawing, cell suspensions were subjected to Ficoll-Isopaque (GE Healthcare) density centrifugation to remove dead cells. In order to assess B cell representation, an aliquot of cells was then stained using the following antibodies: anti-CD38-PE (clone HB7, BD Biosciences), anti-CD19-eFluor 506 (clone HIB19, eBioscience), anti-CD20-Pacific Blue (clone 2H7, BioLegend). Cells were analyzed using the Attune NxT Flow Cytometer (ThermoFisher Scientific) and all specimens showed a fraction of B cells (CD19+/CD20+) >70%, except for BL110-E, in which B cells represented 36% of the total alive cells. Data were analyzed using FlowJo (TreeStar).

Cell suspensions were diluted at a concentration of 1,000 cells/μl and analyzed using the Chromium Next GEM Single Cell 5' kit v2, the Chromium Next GEM Single Cell V(D)J Enrichment Human B cell Kit, and the Chromium Controller (10x Genomics), following the manufacturer's instructions. Sequencing was performed on the NovaSeq 6000 System (Illumina). The FASTQ files were aligned to the human GRCh38 reference genome using the 10x Genomics Cell Ranger software v6.0 to create unique molecular identifier (UMI) count tables of gene expression for each sample using a pipeline that we developed previously[3]. The mRNA libraries from each specimen

displayed a median of 9,045 reads/cell and of 2,275 genes/cell. UMI counts were normalized by library size. To annotate cell contig identifier with V(D)J information, the FASTQ files were aligned to the human GRCh38 reference genome using the Cell Ranger software v6.0 (10x Genomics) and further annotated using the international ImMunoGeneTics information system (IMGT)[4]. Clonally rearranged V(D)J sequences were identified using cells that had good quality expression data and V(D)J information for exactly one heavy and one light chain, with a UMI count >3 and classified by Cell Ranger as productive, full length, and with high confidence annotations. Once the clonal rearrangement was identified, we also included cells that did not fully satisfy the above criteria (i.e. lacking the light or heavy chain) but carried evidence of the clonally rearranged V(D)J sequence.

Data from different patients were merged using the LIGER R package[5]. The merged data were dimensionally reduced using Principal Component Analysis (PCA). UMAP 2D projections were created from the PCA data (parameters: distance='cosine', n_neighbors=30, mist_dist=0.3). The single-cell expression profiles were clustered using the LIGER's non-negative factorization with Louvain clustering. Cluster identification and UMAP predictions in the tumor and non-tumor datasets were performed using Seurat[6]. BL and published sc-transcriptomic profiles of normal and malignant GC B cells were combined and analyzed by Scanpy[7].

The MAST R package was used to test for differentially expressed genes between individual clusters or pairs of clusters[8].

The differences in cell population composition across groups of patients were calculated using non-parametric two-sided Mann-Whitney U Tests.

Exhaustion scores for the T cell subpopulation were calculated using the UCell package[9] using a Mann-Whitney *U* statistic and a predefined exhaustion gene signature to score each cell[10]. A one-sided hypergeometric test was used to assess enrichment or depletion of exhausted cells within patient/group subpopulations.

Pseudo-time analysis was performed using the Slingshot R package[11]. Trajectories were anchored relative to the DZ cells. Multiple lineages were constructed to account for all cells. Since some cells share multiple lineages, we averaged the pseudo-time for a cell across lineages for display purposes.

Tumor cells clusters were labelled using previously identified sc-signatures (100 genes) of normal GC B cells[3]. For each signature, the correlation of the average expression of its genes in each cluster with the signature's normalized expression was calculated. Bootstrapping was then used to measure the correlation between each signature and 100 random genes 1000 times for each cluster to establish a significance for the correlation. Clusters were matched to a signature based on the p-value and highest correlation.

TP53 mutational analysis

Genomic DNA was obtained from tumor tissues at diagnosis using the QIAmp DNA Mini kit (Qiagen), according to the manufacturer's instructions. Mutational analyses were performed on exons 5, 6, 7 and 8 of *TP53* gene. PCR amplification was performed starting from 50ng of genomic DNA using 0.5U of GoTaq Hot Start Polymerase (Promega), 1.5mM $MgCl_2$, 0.2mM of each dNTP and 400nM of each primer. Primer sequences are reported in Supplementary Table 2. For exons 5, 6 and 8, the cycling conditions were as follows: 2' at 94°C; 30'' at 94°C, 45'' at 63°C and 1' at 72°C for 20 cycles; 30'' at 94°C, 45'' at 60°C and 1' at 72°C for 30 cycles; 10' at 72°C. Exon 7 amplification was performed with the following cycling conditions: 2' at 94°C; 30'' at 94°C, 45'' at 60°C and 1' at 72°C for 50 cycles; 10' at 72°C. PCR amplicons were purified using the Illustra ExoProStar 1-Step reagent (Cytiva) and sequenced on a 3500 DX Genetic Analyzer (ThermoFisher Scientific). NM_000546.6 was used as the reference sequence for the mutational analysis.

Cell lines, plasmids, and transient transfection assays

HEK-293T cells were purchased from ATCC, tested for mycoplasma and grown in Dulbecco's Modified Eagle Medium (DMEM) supplemented with 10% fetal calf serum (FCS), 100mg/ml penicillin and streptomycin and maintained at 37°C in a humidified incubator in the presence of 5% CO_2. For transient expression in HEK-293T cells, polyethylenimine (PEI) (Polysciences) was used as previously described[12].

The pCMV-HA-TPM2.1 plasmid was generated by subcloning the PCR-amplified TPM2.1 cDNA (NM_213674) into the pCMV-N-HA vector (Clontech) using SalI and KpnI restriction sites. The plasmid sequence was confirmed by Sanger sequencing.

Immunoblotting

Whole-cell protein extracts were isolated using 50mM Tris-HCl pH 7.5; 200mM NaCl; 0.5% NP-40; 5% glycerol; 2.5mM $MgCl_2$; 0.25% sodium deoxycholate; 0.05% SDS; 1X protease inhibitor cocktail (Sigma-Aldrich) and sonicated in a BioruptorPlus sonicator (Diagenode) for 10min. PAGE was performed using precast 4-20% Tris-glycine gels (Invitrogen) and standard protocols. Following blocking for 1h in 1X phosphate-buffered saline with 0.05% Tween-20 (PBS-T) and 5% non-fat dry milk, the membrane was incubated over-night with anti-TPM2 (1:1,000, rabbit, Proteintech, 11038-1-AP Lot#94880), anti-HA (1:1,000, rat, Roche), and anti-α-Tubulin (1:2,000, mouse, Sigma) antibodies. Detection was performed using horseradish peroxidase-conjugated secondary antibodies (Cytiva) and ECL (Thermo Fisher Scientific) followed by film exposure.

Immunohistochemistry

Formalin-fixed and paraffin-embedded (FFPE) tumor or reactive lymphoid tissue 3μm-thick sections were used for immunohistochemical staining. Tissue sections were deparaffinized and rehydrated before heat-induced epitope retrieval (10mM sodium citrate buffer, pH 6.0 or 10mM Tris-Base, 1mM EDTA, 0.05% Tween20 buffer, pH 9.0). Slides were incubated in 1X PBS with

3% H_2O_2 and the Avidin-Biotin Blocking Kit (Vector Laboratories) and then blocked in PBS-T with 3% bovine serum albumin (BSA; Sigma-Aldrich), followed by overnight incubation at 4°C with anti-TPM2 antibody (1:200, rabbit, Proteintech, 11038-1-AP Lot#94880). Tissue sections were washed and incubated at room temperature with the Envision+ system-HRP rabbit (Dako) for 50min, followed by the AEC substrate (Vector Laboratories) for up to 10min and counterstained with hematoxylin solution (Ricca Chemical Company).

TPM2 expression in BL cases was considered positive when >30% tumor cells showed clear-cut cytoplasmic expression, irrespective of staining intensity. The scoring was performed in sections with reliable internal positive (i.e. blood vessel and/or muscle tissue) and negative (i.e. GC B cells, adipocytes, collagen fibers) controls.

Coverslip-grown transfected HEK-293T cells were fixed in 10% formalin for 10min at RT, permeabilized in 1X PBS with 0.2% Triton-X for 5min blocked in 1X PBS with 3% BSA for 30min and incubated overnight at 4°C with anti-TPM2 (1:200, rabbit, Proteintech, 11038-1-AP Lot#94880) or anti-HA (1:100, rat, Roche) antibodies. After washing, the coverslips were incubated with the Envision+ system-HRP rabbit (Dako) or with goat anti-rat biotinylated secondary antibody (1:200, Southern Biotech) for 50min followed by avidin-HRP (1:200, Dako) for 20min and AEC substrate (Vector Laboratories) for up to 10min.

Images were captured using a DS-Fi3 camera mounted on a NIKON Eclipse E600e microscope (Nikon) and analyzed with NIS-Elements F software (version 5.21.00) and ImageJ (version 1.54d).

Code availability

All analyses and visualizations were performed in R and Python using the following open-source tools as described above: 10x Genomics Cell Ranger software v6.0 (https://support.10xgenomics.com/single-cell-gene-expression/software/pipelines/latest/installation), LIGER (https://github.com/welch-lab/liger),

Seurat v4.1 (https://satijalab.org/seurat/), Scanpy (https://scanpy.readthedocs.io/en/stable/), MAST (https://bioconductor.org/packages/release/bioc/html/MAST.html), DESeq2 (https://bioconductor.org/packages/release/bioc/html/DESeq2.html), the Scikit-learn implementations of PCA, Lifelines (https://github.com/CamDavidsonPilon/lifelines/), Seaborn (https://seaborn.pydata.org/index.html), and GSEA (https://www.gsea-msigdb.org/gsea/index.jsp). Figures were partially created with the svgwrite package (https://pypi.org/project/svgwrite/).

REFERENCES

1 Pillon, M., Mussolin, L., Carraro, E., Conter, V., Arico, M., Vinti, L., Garaventa, A., Piglione, M., Buffardi, S., Sala, A., Santoro, N., Lo Nigro, L., Mura, R., Tondo, A., Casale, F., Farruggia, P., Pierani, P., Cesaro, S., d'Amore, E. S. & Basso, G. Detection of prognostic factors in children and adolescents with Burkitt and Diffuse Large B-Cell Lymphoma treated with the AIEOP LNH-97 protocol. *Br J Haematol* **175**, 467-475 (2016). 10.1111/bjh.14240

2 Murphy, S. B. Classification, staging and end results of treatment of childhood non-Hodgkin's lymphomas: dissimilarities from lymphomas in adults. *Semin Oncol* **7**, 332-339 (1980).

3 Holmes, A. B., Corinaldesi, C., Shen, Q., Kumar, R., Compagno, N., Wang, Z., Nitzan, M., Grunstein, E., Pasqualucci, L., Dalla-Favera, R. & Basso, K. Single-cell analysis of germinal-center B cells informs on lymphoma cell of origin and outcome. *J Exp Med* **217** (2020). 10.1084/jem.20200483

4 Lefranc, M. P., Giudicelli, V., Duroux, P., Jabado-Michaloud, J., Folch, G., Aouinti, S., Carillon, E., Duvergey, H., Houles, A., Paysan-Lafosse, T., Hadi-Saljoqi, S., Sasorith, S., Lefranc, G. & Kossida, S. IMGT(R), the international ImMunoGeneTics information system(R) 25 years on. *Nucleic Acids Res* **43**, D413-422 (2015). 10.1093/nar/gku1056

5 Welch, J. D., Kozareva, V., Ferreira, A., Vanderburg, C., Martin, C. & Macosko, E. Z. Single-Cell Multi-omic Integration Compares and Contrasts Features of Brain Cell Identity. *Cell* **177**, 1873-1887 e1817 (2019). 10.1016/j.cell.2019.05.006

6 Butler, A., Hoffman, P., Smibert, P., Papalexi, E. & Satija, R. Integrating single-cell transcriptomic data across different conditions, technologies, and species. *Nat Biotechnol* **36**, 411-420 (2018). 10.1038/nbt.4096

7 Liu, N., Jiang, C., Yao, X., Fang, M., Qiao, X., Zhu, L., Yang, Z., Gao, X., Ji, Y., Niu, C., Cheng, C., Qu, K. & Lin, J. Single-cell landscape of primary central nervous system diffuse large B-cell lymphoma. *Cell Discov* **9**, 55 (2023). 10.1038/s41421-023-00559-7

8 Finak, G., McDavid, A., Yajima, M., Deng, J., Gersuk, V., Shalek, A. K., Slichter, C. K., Miller, H. W., McElrath, M. J., Prlic, M., Linsley, P. S. & Gottardo, R. MAST: a flexible statistical framework for assessing transcriptional changes and characterizing heterogeneity in single-cell RNA sequencing data. *Genome Biol* **16**, 278 (2015). 10.1186/s13059-015-0844-5

9 Andreatta, M. & Carmona, S. J. UCell: Robust and scalable single-cell gene signature scoring. *Comput Struct Biotechnol J* **19**, 3796-3798 (2021). 10.1016/j.csbj.2021.06.043

10 Roider, T., Baertsch, M. A., Fitzgerald, D., Vohringer, H., Brinkmann, B. J., Czernilofsky, F., Knoll, M., Llao-Cid, L., Mathioudaki, A., Fassbender, B., Herbon, M., Lautwein, T., Bruch, P. M., Liebers, N., Schurch, C. M., Passerini, V., Seifert, M., Brobeil, A., Mechtersheimer, G., Muller-Tidow, C., Weigert, O., Seiffert, M., Nolan, G. P., Huber, W. & Dietrich, S. Multimodal and spatially resolved profiling identifies distinct patterns of T cell infiltration in nodal B cell lymphoma entities. *Nature cell biology* **26**, 478-489 (2024). 10.1038/s41556-024-01358-2

11 Street, K., Risso, D., Fletcher, R. B., Das, D., Ngai, J., Yosef, N., Purdom, E. & Dudoit, S. Slingshot: cell lineage and pseudotime inference for single-cell transcriptomics. *BMC Genomics* **19**, 477 (2018). 10.1186/s12864-018-4772-0

12 Ying, C. Y., Dominguez-Sola, D., Fabi, M., Lorenz, I. C., Hussein, S., Bansal, M., Califano, A., Pasqualucci, L., Basso, K. & Dalla-Favera, R. MEF2B mutations lead to deregulated expression of the oncogene BCL6 in diffuse large B cell lymphoma. *Nat Immunol* **14**, 1084-1092 (2013). 10.1038/ni.2688
