# Supplementary material for: Single-cell transcriptomics of pediatric Burkitt lymphoma reveals intra-tumor heterogeneity and markers of therapy resistance
Source: Leukemia. 2024 Oct 18;39(1):189–98. doi: 10.1038/s41375-024-02431-3 (PMC11717704; doi:10.1038/s41375-024-02431-3)
Supplement: Supplementary file 2 — Supplementary Figures [file 41375_2024_2431_MOESM2_ESM.pdf]

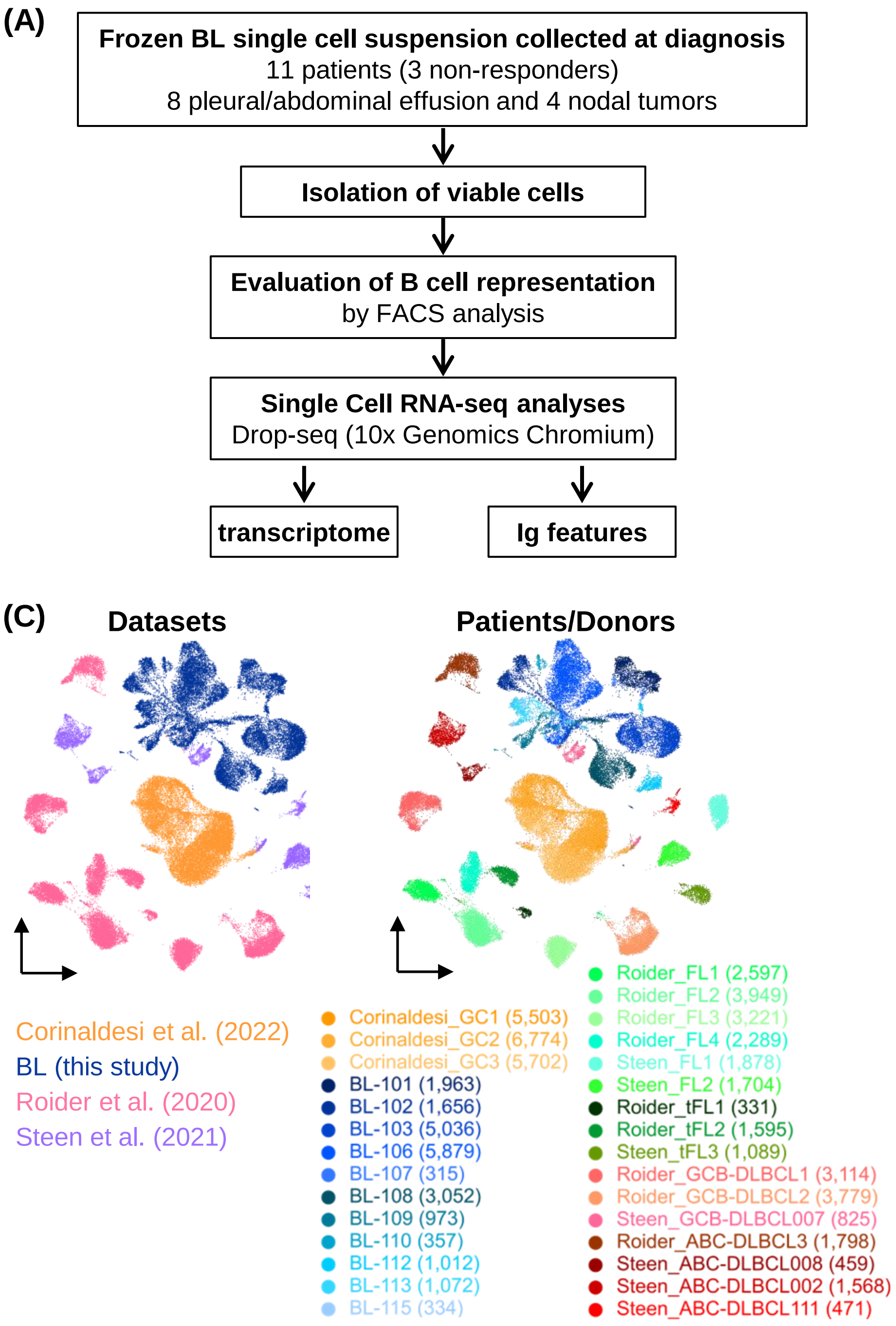

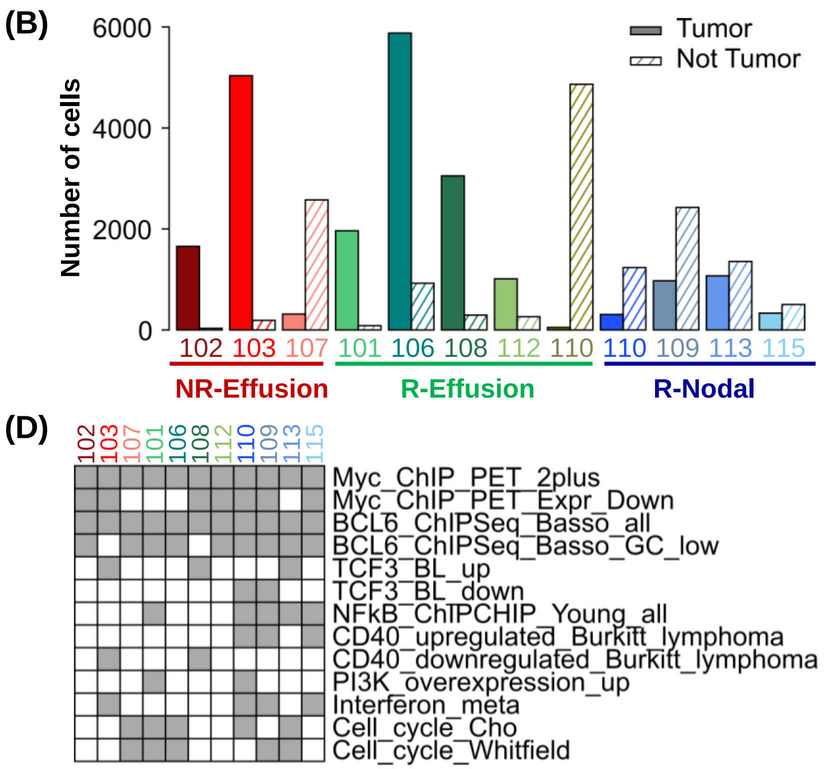

Supplemental Figure 1. Single-cell transcriptomic analysis of Burkitt Lymphoma. (A) Schematic flowchart summarizing the experimental procedure. **(B)** Histogram bar plot depicting the number of tumor cells (solid colors) and tumor-infiltrating normal cells (not tumor, striped colors) in each of the specimens analyzed by sc-transcriptomics. Specimens are grouped based on the sample origin, as effusion or tumor mass (nodal), and on the response to therapy (non-responders, NR; responders, R). **(C)** UMAP projection of sc-transcriptomic profiles of normal GC B cells and tumor cells isolated from BL, follicular lymphoma (FL), transformed-FL (tFL), diffuse large B cell lymphoma (DLBCL), including both GCB and ABC subtypes**.** UMAPs are color-coded according to the dataset or patient/donor origin. The immunoglobulin gene transcripts are not included. The number of cells is provided in parenthesis. **(D)** Pathway enrichment analysis for the gene signatures (top 100 upregulated genes) associated with each patient. Relevant pathways from the SignatureDB database that are significantly enriched (hypergeometric test with Benjamini-Hochberg correction, q<0.05) are shown in grey.

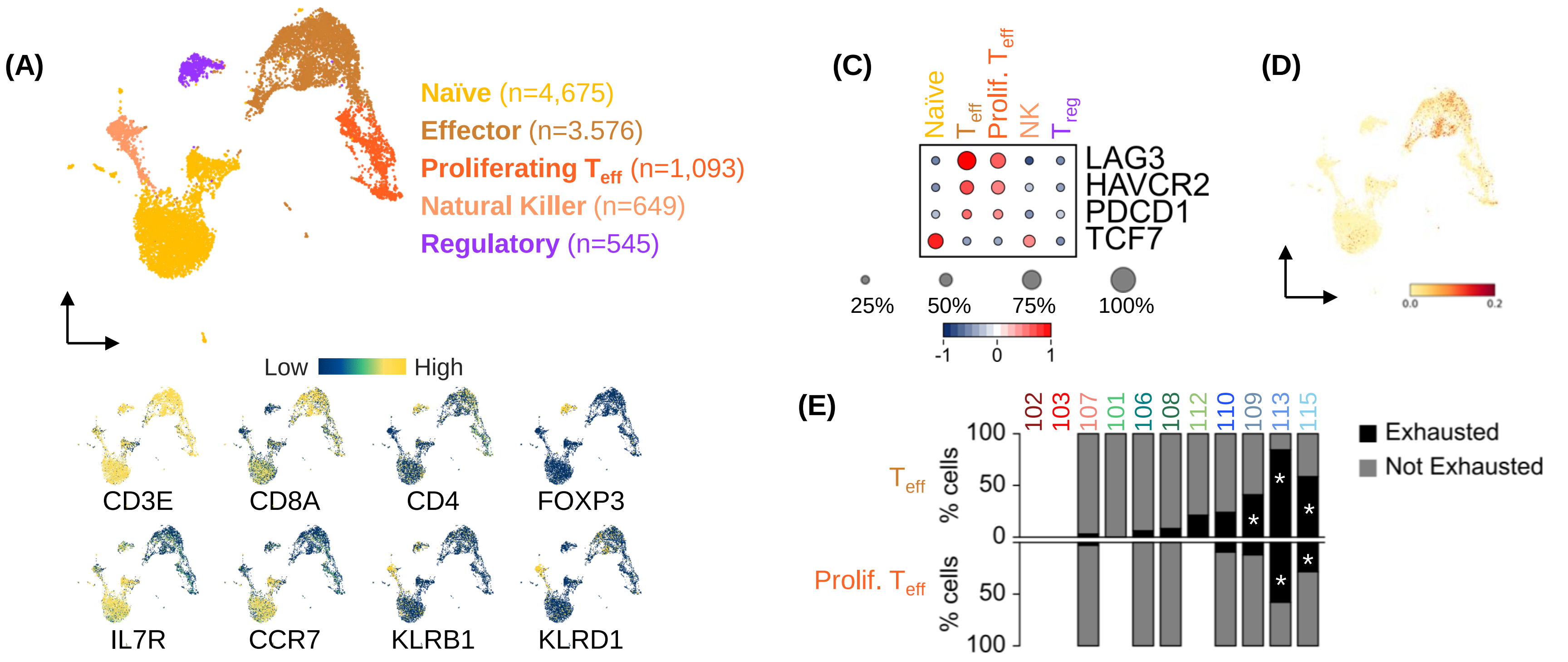

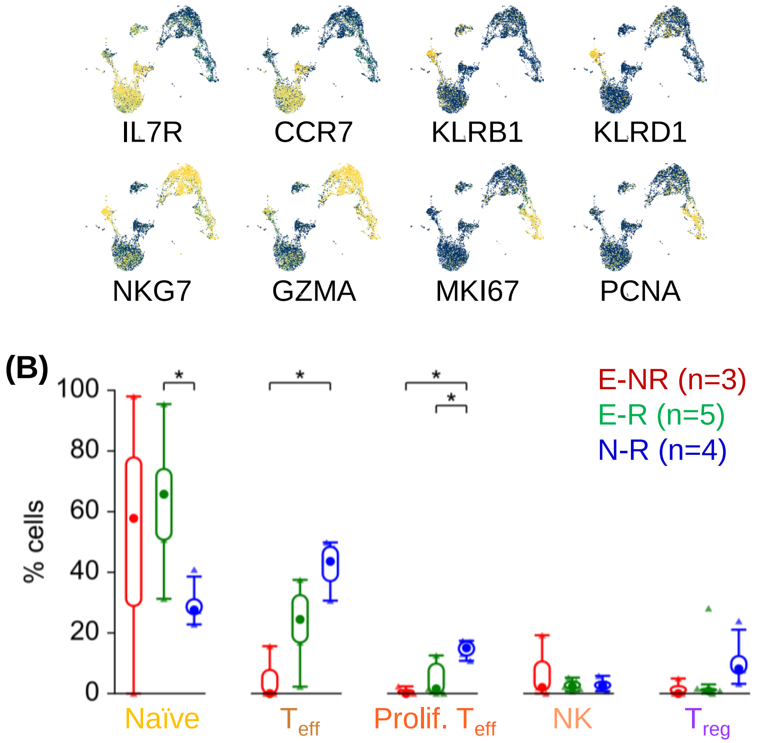

Supplemental Figure 2. Single-cell transcriptomic analysis of infiltrating normal T cells in BL specimens. (A) UMAP projection of sc-transcriptomic profiles of 10,538 tumor-infiltrating normal T cells obtained from the merge of all specimens, upon batch correction. Cells are labeled based on the identified populations. (Bottom) Relative expression of transcripts of interest associated with the distinct populations is displayed as UMAP/heatmap with colors representing the z-scored log2 normalized expression. **(B)** Whisker box plots displaying the distribution of tumor-infiltrating normal T cells in the dataset stratified by specimen type (effusions, E vs nodal tumor, N) and response to therapy (non-responders, NR; responders, R). A Mann-Whitney U Test was used to compare data sets pairwise. Significant associations ($p<0.05$) are indicated with an asterisk. **(C)** Heat map displaying relative expression of exhaustion marker genes. The size of the dot indicates the percentage of cells with detectable expression, and the color reflects the relative gene expression shown as the z-scored average (log2) normalized expression within a group. **(D)** UMAP/heat map of exhaustion score. **(E)** Percentages of exhausted (exhaustion score >0) T_{eff} and Proliferating T_{eff} cells across patients. Asterisk indicates significant enrichment ($p<0.05$).

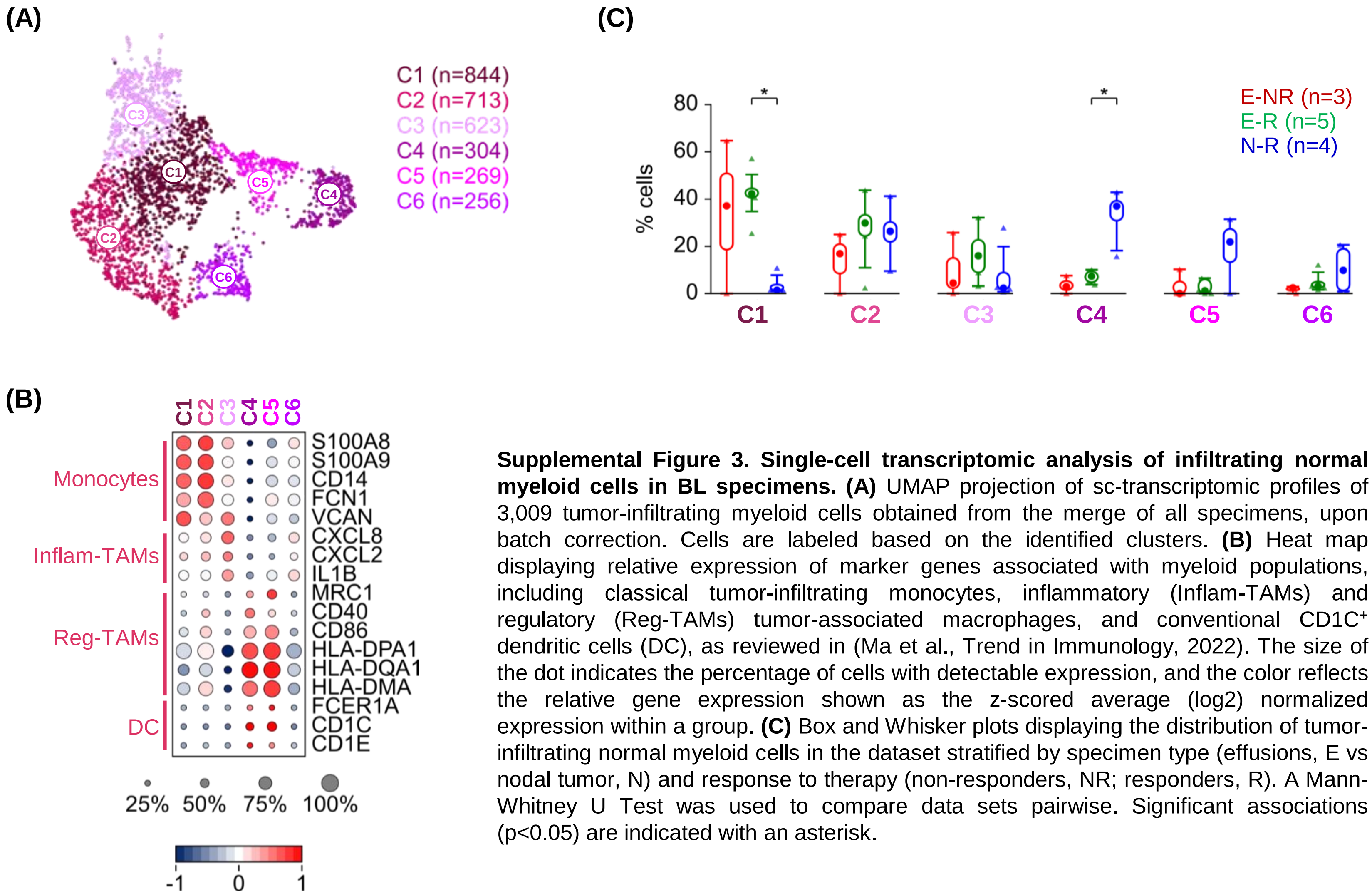

Supplemental Figure 3. Single-cell transcriptomic analysis of infiltrating normal myeloid cells in BL specimens. (A) UMAP projection of sc-transcriptomic profiles of 3,009 tumor-infiltrating myeloid cells obtained from the merge of all specimens, upon batch correction. Cells are labeled based on the identified clusters. **(B)** Heat map displaying relative expression of marker genes associated with myeloid populations, including classical tumor-infiltrating monocytes, inflammatory (Inflam-TAMs) and regulatory (Reg-TAMs) tumor-associated macrophages, and conventional CD1C^{+} dendritic cells (DC), as reviewed in (Ma et al., Trend in Immunology, 2022). The size of the dot indicates the percentage of cells with detectable expression, and the color reflects the relative gene expression shown as the z-scored average (log2) normalized expression within a group. **(C)** Box and Whisker plots displaying the distribution of tumor-infiltrating normal myeloid cells in the dataset stratified by specimen type (effusions, E vs nodal tumor, N) and response to therapy (non-responders, NR; responders, R). A Mann-Whitney U Test was used to compare data sets pairwise. Significant associations ($p<0.05$) are indicated with an asterisk.

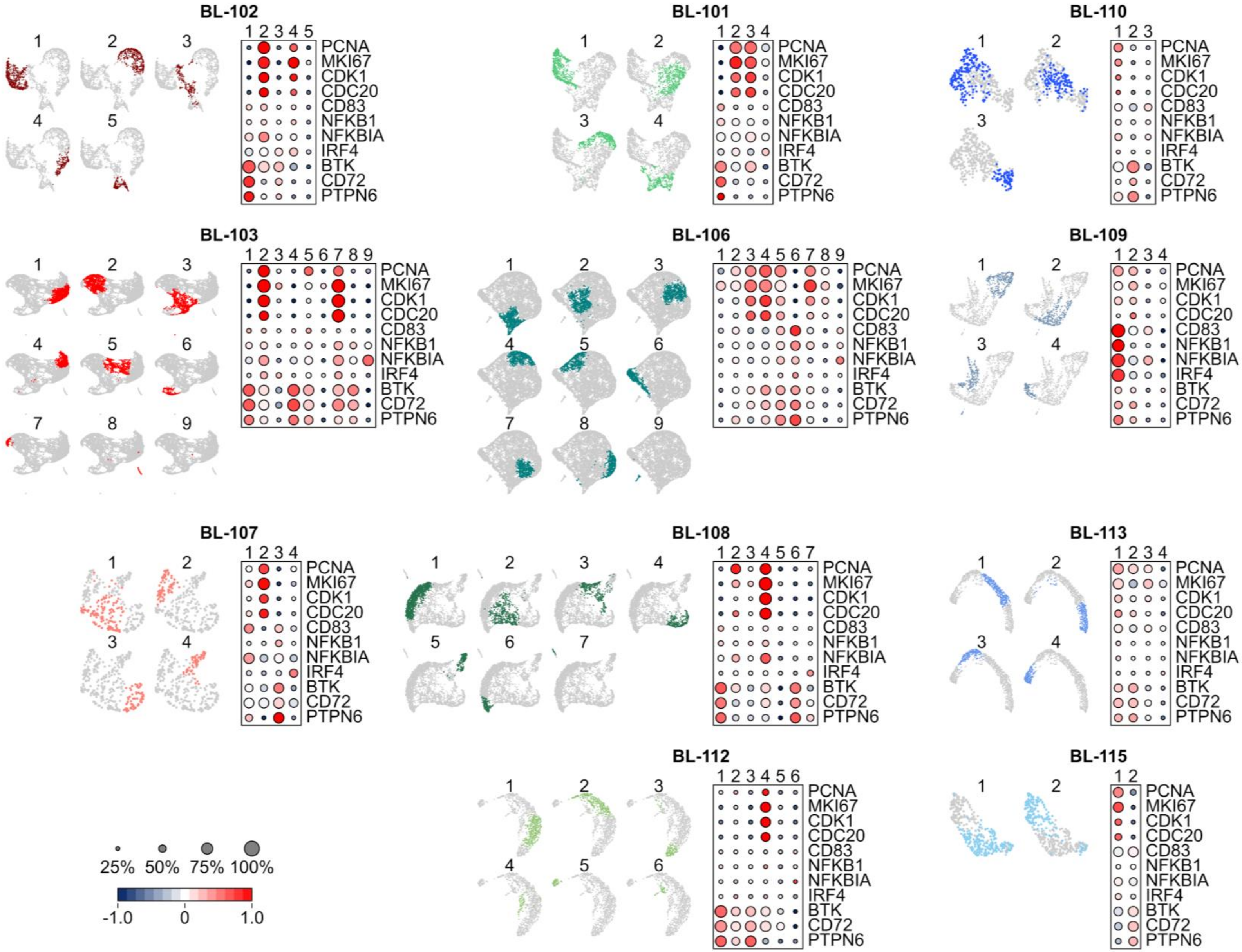

Supplemental Figure 4. BL tumor cells display a heterogeneous transcriptome. UMAP projection and cluster identification by LIGER in BL tumor cells from each of the 11 BL patients. In the UMAPs shades of red are used for diagnostic specimens obtained from non-responders, while green (effusion specimens) and blue (nodal specimens) are used for therapy responders. Heat maps display expression in each cluster for a subset of differentially expressed genes. The size of the dot indicates the percentage of cells with detectable expression, and the color reflects the relative gene expression shown as the z-scored average (log2) normalized expression within a cluster.

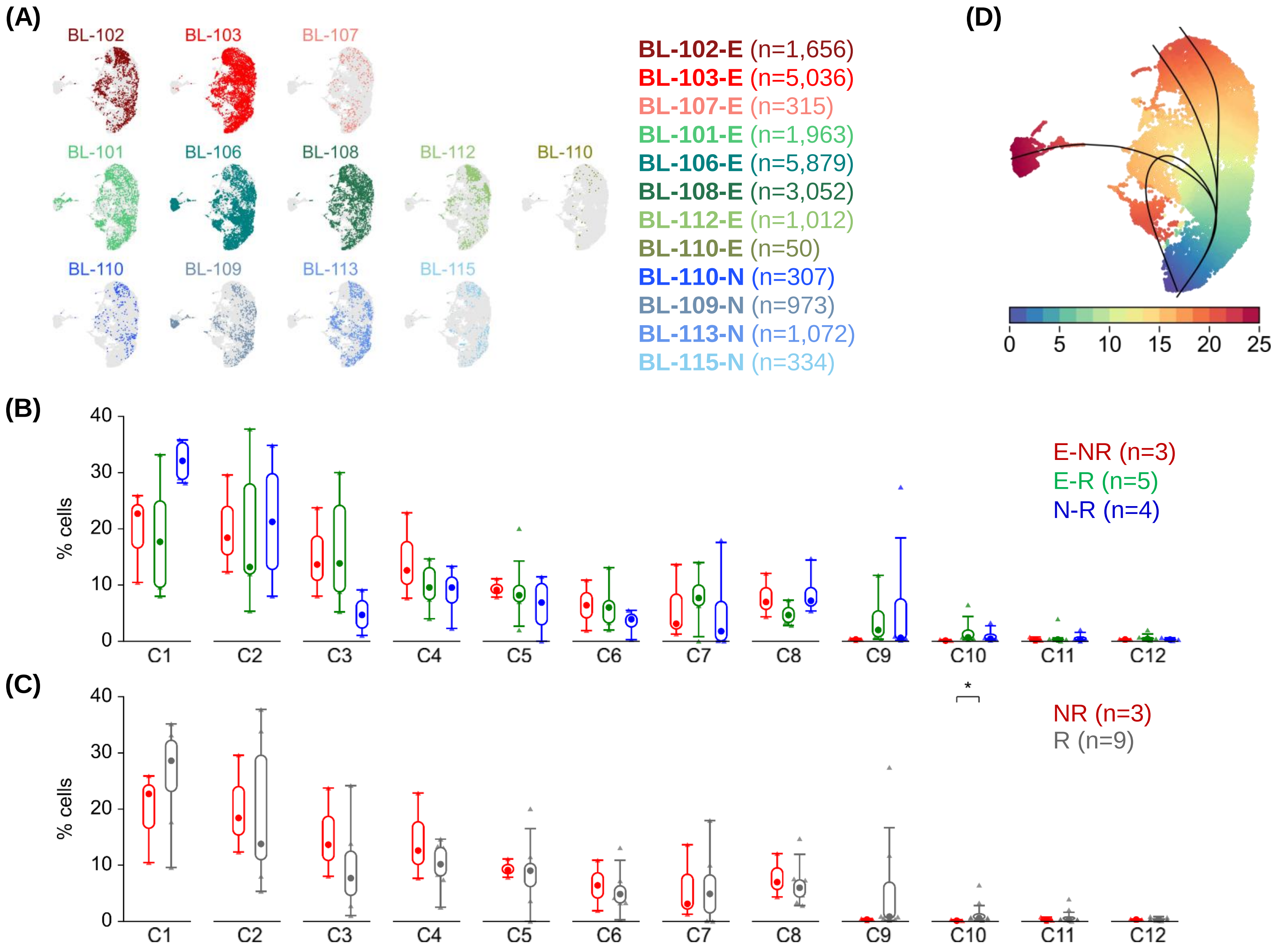

Supplemental Figure 5. BL intra-tumor heterogeneity is shared across patients. (A) The UMAP projection of sc-transcriptomic profiles of 21,649 BL tumor cells obtained by merging all specimens, upon batch correction, is color-coded based on the cells contributed by each patient. **(B-C)** Box and Whisker plots displaying the distribution across the clusters of tumor cells stratified by **(B)** specimen type (effusions vs nodal), and **(C)** response to therapy (non-responders, NR; responders, R). **(D)** The trajectories inferred by pseudo-time analysis were overlayed on the UMAP of BL tumor cells. The color bar represents pseudo-time on an arbitrary scale.

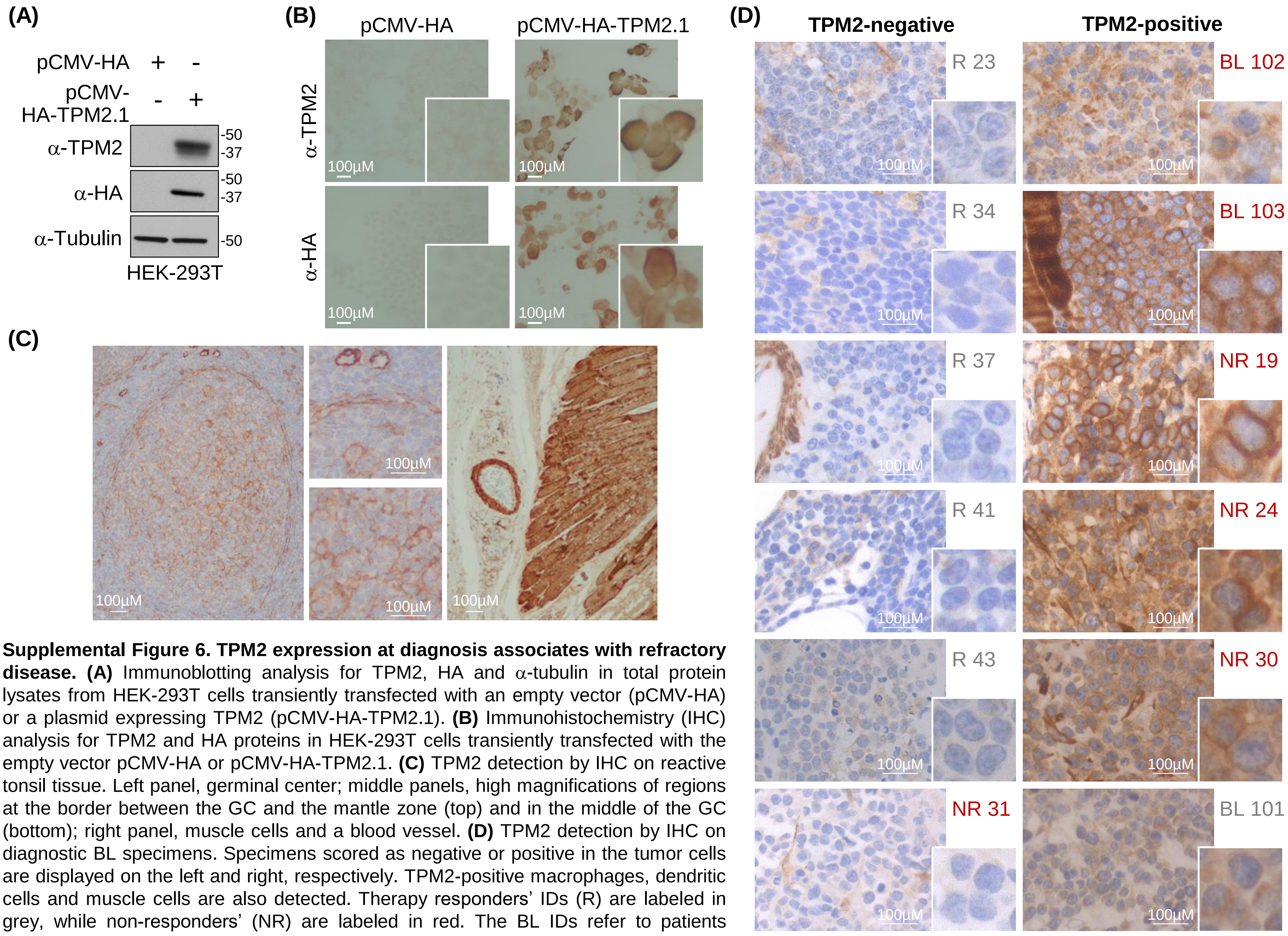

Supplemental Figure 6. TPM2 expression at diagnosis associates with refractory disease. (A) Immunoblotting analysis for TPM2, HA and α-tubulin in total protein lysates from HEK-293T cells transiently transfected with an empty vector (pCMV-HA) or a plasmid expressing TPM2 (pCMV-HA-TPM2.1). **(B)** Immunohistochemistry (IHC) analysis for TPM2 and HA proteins in HEK-293T cells transiently transfected with the empty vector pCMV-HA or pCMV-HA-TPM2.1. **(C)** TPM2 detection by IHC on reactive tonsil tissue. Left panel, germinal center; middle panels, high magnifications of regions at the border between the GC and the mantle zone (top) and in the middle of the GC (bottom); right panel, muscle cells and a blood vessel. **(D)** TPM2 detection by IHC on diagnostic BL specimens. Specimens scored as negative or positive in the tumor cells are displayed on the left and right, respectively. TPM2-positive macrophages, dendritic cells and muscle cells are also detected. Therapy responders' IDs (R) are labeled in grey, while non-responders' (NR) are labeled in red. The BL IDs refer to patients analyzed also by sc-transcriptomic.
