# Supplementary material for: Single-cell transcriptomics of pediatric Burkitt lymphoma reveals intra-tumor heterogeneity and markers of therapy resistance
Source: Leukemia. 2024 Oct 18;39(1):189–98. doi: 10.1038/s41375-024-02431-3 (PMC11717704; doi:10.1038/s41375-024-02431-3)
Supplement: Supplementary file 4 — Supplementary Table 2 [file 41375_2024_2431_MOESM4_ESM.pdf]

Supplementary Table 2. Sequences of primers used for quantitative Real-time PCR and *TP53* mutational analysis.

**qRT-PCR primer sequences**	
GAPDH_FOR	5'- TCCTCTGACTTCAACAGCGA -3'
GAPDH_REV	5'- GGGTCTTACTCCTTGGAGGC -3'
TPM2_FOR	5'- AGCTGGAGGAGGAGCAGCA-3'
TPM2_REV	5'- TCAGCATCAGTGGCCTTCTTC-3'
CD72_FOR	5'- CTTACTACTTCTTAAATTCACTGTTGCCA-3'
CD72_REV	5'- AAGTCCTAGTGCGTTGTGTATC-3'
SOX11_FOR	5'- CAGCAAGAAATGCGGCAA-3'
SOX11_REV	5'- GACGACTACGTGCTGGGCA-3'
MYB_FOR	5'- CTGGTGGAACAGAATGGAACAGA-3'
MYB_REV	5'- CCTTGATGAGCTCAGGGTTTAGTAC-3'
BTK_FOR	5'- ATCCACAGGGGACCCTCAAG-3'
BTK_REV	5'- GCCTGGATATGAGTCCTGCAG-3'

**TP53 mutational analysis primer sequences**	
Exon 5_FOR	5’-TTCACTTGTGCCCTGACTTTCA-3’
Exon 5_REV	5’-CAGCCCTGTCGTCTCTCCAG-3’
Exon 6_FOR	5’-GCCTCTGATTCCTCACTGAT-3’
Exon 6_REV	5’- TTAACCCCTCCTCCCAGAGA-3’
Exon 7_FOR	5’-CTTGCCACAGGTCTCCCCAA-3’
Exon 7_REV	5’- AGGGGTCAGAGGCAAGCAGA-3’
Exon 8_FOR	5’-TTCCTTACTGCCTCTTGCTT-3’
Exon 8_REV	5’- AGGCATAACTGCACCCTTGG-3’
