# Supplementary material for: Single-cell transcriptomics of pediatric Burkitt lymphoma reveals intra-tumor heterogeneity and markers of therapy resistance
Source: Leukemia. 2024 Oct 18;39(1):189–98. doi: 10.1038/s41375-024-02431-3 (PMC11717704; doi:10.1038/s41375-024-02431-3)
Supplement: Supplementary file 5 — Supplementary Table 3 [file 41375_2024_2431_MOESM5_ESM.pdf]

**Supplementary Table 3.** Clonal rearrangements for the heavy (IGH) and light (IGL) immunoglobulin chains, as detected by single cell V(D)J analysis.

| Patient ID | IGHV | IGHC | IGL |
|---|---|---|---|
| BL_102 | IGHV3-20,IGHJ4 | IGHM | IGKV3-20,IGKJ2 |
| BL_103 | IGHV2-26,IGHD2-15,IGHJ5 | IGHM/D | IGLV3-1,IGLJ2 |
| BL_107 | IGHV3-7,IGHJ4 | IGHM | IGKV2-30,IGKJ3 |
| BL_101 | IGHV3-23,IGHJ4/5 | IGHM | IGKV3-15,IGKJ4 |
| BL_106 | IGHV3-23,IGHJ6 | IGHM/D | IGLV3-21,IGLJ1 |
| BL_108 | IGHV3-33,IGHJ4 | IGHA1/M | IGLV2-14,IGLJ1 |
| BL_112 | IGHV4-39,IGHD3-22,IGHJ4 | IGHM | IGLV1-51,IGLJ2 |
| BL_110 | IGHV3-33,IGHJ3 | IGHM/D | IGLV2-14,IGLJ2 |
| BL_109 | IGHV1-18,IGHJ4 | IGHM | IGKV1-33,IGKJ4 |
| BL_113 | IGHV4-59,IGHD3-22,IGHJ4 | IGHM | IGLV1-51,IGLJ2 |
| BL_115 | IGHV3-64D,IGHJ4 | IGHM | IGLV2-8,IGLJ2 |
